# Non-Destructive Genotyping of Cultivars and Strains of Sesame through NIR Spectroscopy and Chemometrics

**DOI:** 10.3390/bios12020069

**Published:** 2022-01-26

**Authors:** Francisco dos Santos Panero, Oscar Smiderle, João S. Panero, Fernando S. D. V. Faria, Pedro dos S. Panero, Anselmo F. R. Rodriguez

**Affiliations:** 1 Chemistry Department, Federal University of Roraima, Boa Vista 69310-000, RR, Brazil; 2 Brazilian Agricultural Research Corporation (Embrapa), Boa Vista 69301-970, RR, Brazil; oscar.smiderle@embrapa.br; 3 Federal Institute of Science and Technology of Roraima, Campus Boa Vista, Boa Vista 69303-340, RR, Brazil; joao.panero@ifrr.edu.br; 4 Science and Technology Innovation Program, Federal University of Acre, Rio Branco 69915-900, AC, Brazil; fernandoescocio@hotmail.com (F.S.D.V.F.); anselmo.rodriguez@ufac.br (A.F.R.R.); 5 Biodiversity and Biotechnology Network of the Legal Amazon, Biotechnology Department, Federal University of Acre, Rio Branco 69915-900, AC, Brazil; 6 Federal Institute of Science and Technology of Roraima, Campus Zona Oeste, Boa Vista 69318-000, RR, Brazil; pedro.panero@ifrr.edu.br

**Keywords:** *Sesamum indicum*, NIR, PCA, HCA, KNN, SIMCA

## Abstract

The differentiation of cultivars is carried out by means of morphological descriptors, in addition to molecular markers. In this work, near-infrared spectroscopy (NIR) and chemometric techniques were used to develop classification models for two different commercial sesame cultivars (*Sesamum indicum*) and 3 different strains. The diffuse reflectance spectra were recorded in the region of 700 to 2500 nm. Based on the application of chemometric techniques: principal component analysis—PCA, hierarchical cluster analysis—HCA, k-nearest neighbor—KNN and the flexible independent modeling of class analogy—SIMCA, from the infrared spectra in the near region, it was possible to perform the genotyping of two sesame cultivars (BRS Seda and BRS Anahí), and to classify these cultivars with 3 different sesame strains, obtaining 100% accurate results. Due to the good results obtained with the implemented models, the potential of the methods for a possible realization of forensic, fast and non-destructive authentication, in intact sesame seeds was evident.

## 1. Introduction

The seed *Sesamum indicum* L. is known in Brazil as sesame, belonging to the Pedalium family; it is an annual or perennial herbaceous plant. Depending on the cultivar, it is one of the oldest oilseeds in use by man, and there are records of its cultivation more than 4300 years before Christ. In Brazil, it was introduced by the Portuguese in the 16th century [1,2].

Sesame is considered as the ninth most cultivated oilseed in the world and an oilseed of great economic importance. It is grown in more than 70 countries, especially on the Asian and African continents, with India, Myanmar and China accounting for 51.96% of world production [3,4].

Sesame seeds are small and flattened, and can present varied colors, ranging from white to black, passing through to brown and golden yellow [2]. Sesame can be used in the food, chemical and pharmaceutical industries. Its seeds contain over 50% of excellent quality oil, reaching up to 63% in some varieties [2,5].

Sesame grains are consumed both fresh and in various culinary preparations. Due to the new ways of using sesame seeds and their by-products, their economic importance has been gradually growing. The sesame market is growing, especially in the bakery and biscuit sectors [6].

Sesame has been used in the food industry, mainly in baking, especially for cookies and sweets, and due to this use in baking, its consumption has been leveraged; since then it has grown at a rate of 10% per year. Its main crop still remains for oil production [3].

Seeds and grains are used as raw material for the food industry. Several researches have been carried out in order to find or even develop seeds of a good quality and improve cultivars capable of providing increased productivity. Some cultivars are improved to record pests, greater productivity and/or greater nutritional value [7]. For this reason, they have the right to intellectual property that can be of a high market value [8]. The identification of cultivars is of fundamental importance in the quality control of seeds and grains, due to the growing need to protect them [9].

Sesame seeds have several varieties and cultivars, which have characteristics for specific needs, such as the resistance to pests and edaphoclimatic conditions [10]. A good and new cultivar must have characteristics different from the others existing ones, and those characteristics must remain for successive generations [11,12,13]. 

The differentiation of cultivars is performed by means of morphological descriptors, in addition to molecular markers, which is performed by the study of DNA [10]. Among these markers, the most used are: RFLP (restriction fragment length polymorphism), AFLP (amplified fragment length polymorphism), RAPD (random amplification of polymorphic DNA) and microsatellites (ISSR—inter simple sequence repeats; SSR—simple sequence repeats) [12,14]. In general, the procedure for identifying some cultivars is carried out by planting the seed and, at least, one month is expected to pass so that, through their growth and development, their morphological identification occurs [15]. 

The molecular analysis method also has the disadvantages of being destructive and requiring expensive reagents that generate waste. In addition, these techniques require professionals with technical qualifications [10].

Infrared spectroscopy in the NIR region is considered a powerful tool for the quantitative and qualitative analysis of chemical and physical variables, and can be applied to samples of various types, such as from the pharmaceutical, polymer, petrochemical, food and agricultural industries [15,16]. However, in order to obtain results from the interactions with the analyte (seeds and grains), the development of chemometric models is required [12,17]. These techniques refer to mathematical models and methods and, among these, multivariate statistics, which consider the correlation between many variables analyzed simultaneously, allowing the extraction of a much greater amount of information.

Therefore, the techniques for evaluating the quality of seeds and grains, quickly and non-destructively, can be applied for their selection and classification, mainly because it is a food matrix, whose composition presents a high variability, being influenced by the variety or cultivar, climatic conditions, soil and industrial processing [10,13].

Recent studies have demonstrated the potential of near-infrared (NIR) for the discrimination of seeds, namely non-destructive phenotyping using near-infrared spectroscopy developed classification models for two different commercial castor cultivars using PCA and SIMCA [15]; the separation of 4 soybean cultivars by near-infrared spectroscopy using PCA and HCA [18]; Fourier transform near-infrared spectroscopy combined with discriminant analyses was successfully utilized to classify two cultivars of sweetcorn seeds, based on the full-range wavelengths (1000–2500 nm) [19]; near-infrared spectroscopy and supervised pattern recognition techniques were used to classify five different macadamia cultivars based on intact nuts [20]; a near infrared (NIR) spectroscope as well as ED-XRF were used for the non-destructive discrimination of sesame seed origins (Korean, Chinese and Indian) [21]; and a discriminatory predictive model was used to determine the geographic origin of sesame seeds from Korea, China and other countries (India, Nigeria and Ethiopia) by NMR-based metabolomics focusing on polar metabolites [22].

The scientific literature shows the application of NIR to classify the geographic origin of sesame seeds; however, the chemical composition of the soil varies, as well as the climatic conditions. Some scientists performed the classification of cultivars through NIR in seeds cultivated in the same soil and the same climatic conditions, which some call “NIR phenotyping”.

Therefore, this study aims to demonstrate the classification of sesame cultivars through intact seeds from the same soil and climatic conditions, using the unsupervised pattern recognition techniques, PCA and HCA. Additionally, the development of sesame seed cultivar and strain classification models were analyzed using the machine learning techniques KNN and SIMCA, in spectra in the near-infrared regions, in what we could call “NIR genotyping”.

## 2. Materials and Methods

### 2.1. Samples

For this study, samples of 2 cultivars and 3 different sesame strains were used, provided by the Brazilian Agricultural Research Corporation (Embrapa) in the city of Boa Vista, State of Roraima. All sesame varieties were cultivated by Embrapa, at the *Água Boa* Experimental Field, under the same conditions and soil type, harvested in the mature stage, stored at 25 ± 3 °C at approximately 75% relative humidity. The cultivars were BRS Seda and BRS Anahí, and the strains were 5, 16 and 17.

### 2.2. Near Infrared Spectroscopy

A total of 45 glass bottles containing seed samples of each cultivar and strain (without treatment) were used; only pieces of the plants, leaves and other interferences were removed before the acquisition of the spectra. The diffuse reflectance spectra obtained from the infrared in the near-NIR region of the samples of sesame seeds were obtained at the Nanobiotechnology Laboratory of the Bionorte Complex—UFAC, using the “Spectrum Two FT-IR” spectrophotometer from PerkinElmer, with the diffuse reflectance accessory in the NIR, in the spectral range of 14000 to 4000 cm^−1^ (700 to 2500 nm). The spectra were acquired with a resolution of 8 cm^−1^ and an average spectrum of 50 scans in 30 s.

**Figure 1:**
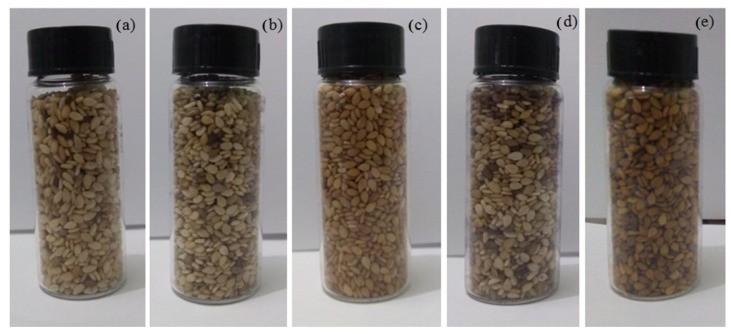
Sesame samples—cultivars: (**a**) BRS Anahí and (**b**) BRS Seda; strains: (**c**) LinGe16, (**d**) LinGe17 and (**e**) LinGe 5 in intact seeds, in a 20 mL glass bottle, for spectra acquisition.

For the acquisition of spectra, the sesame seed samples, (a) BRS Anahí, (b) BRS Seda, (c) strain 16, (d) strain 17 and (e) strain 5, were placed, whole, in 20 mL glass bottles with uniform, transparent walls and backgrounds; samples from the 45 glass bottles containing each cultivar and strain are shown in Figure 1.

### 2.3. Data Processing and Statistical Analysis

The hierarchical cluster analysis—HCA and the principal component analysis—PCA are used to create graphs that represent the largest possible amount of information contained in a set of analytical data. 

PCA is considered one of the unsupervised pattern recognition (PR) methods that are used to examine the similarities or differences between the samples [23]. It is used to maximize the information that can be extracted from a set of spectroscopic data, as it correlatively transforms an original set of variables into a smaller set of variables that contain most of the information from the original set. In this way, it reduces the size of the data in order to generate new variables that are not correlated.

HCA is a method of clustering analysis that is unsupervised, hierarchical and agglomerative, which aims to build a division of groups, in which samples or variables are grouped together in a hierarchical way from the closest (similar) to the most distant, being then expressed in a tree structure (dendrogram) [24].

The matrix used in the PCA and HCA was composed of the spectral mean of the 45 glass bottles, totaling 225 lines (samples) and 4859 columns (wavelength).

Supervised pattern recognition techniques, also called classification methods, are techniques used in machine learning, which is a subfield of artificial intelligence. The classification methods, mostly used in the file of Chemistry, are the algorithms of the nearest neighbor (k-nearest neighbor—KNN) and the flexible independent modeling of class analogy (SIMCA) [25].

KNN is a supervised, non-parametric, discriminating and deterministic pattern recognition algorithm. During the model construction process, each sample from the calibration set (training) was excluded, and later classified using the remaining samples in the training set, with this sample being excluded only once. Thus, cross-validation is already performed simultaneously during the construction of the classification model [26].

One of the most used tools to check the accuracy of a classification is the error matrix, also known as a consistency matrix or a consistency confusion matrix. It is a square matrix, (*n* × *n*), where *n* is the number of classes in which the columns express the prediction errors and successes, and the lines are the classifiers. The main diagonal lists the samples correctly classified [24].

SIMCA is a supervised, parametric, probabilistic and modeling pattern recognition algorithm, and a PCA-based technique, which models the multidimensional space formed by the samples for class definition [27]. As each category in SIMCA is modeled independently using the PCA; the number of main components for each class is calculated through cross-validation in the calibration set.

SIMCA allows independent modeling, where each class is modeled separately and is thus not influenced by samples from other classes; this is very useful for updating data and models, as this method allows for the insertion of new classes into the model without changing the classes that have already been modeled [28]. 

The matrix used in SIMCA and KNN was composed of the spectral average of the 45 glass bottles, for which 30 glass bottles of each cultivar and strain were used, generating a calibration set with 150 samples. In the external validation set, 15 glass bottles of each cultivar and strain were used, generating an external forecast set with 75 samples.

Spectra were obtained from the bottom of the flasks. Each spectrum was saved in the “Spectrum” program, in JCAMPDX format, then imported into the computer software The Unscrambler 9.2 for assembling the spectral matrices with 45 lines (samples) and 4859 columns (wavelength) in the case of sesame spectra. The spectral matrices were assembled and transferred to the Pirouette 3.11 software, which was used for the application of several chemometric methods, such as PCA, HCA, KNN and SIMCA. 

To reduce the effect of noise, remove redundant information and enhance the sample-to-sample differences, the rise in the baseline and the effect of light mirroring due to diffuse reflectance, through some mathematical methods, such as the first and second derivative, SNV (normal signal standardization), MSC (multiplicative signal correction), EMSC (extended multiplicative scatter correction), moving average, Savitzky–Golay, among other techniques and their combinations.

## 3. Results and Discussion

### Authentification of the Cultivars and Sesame Strain

**Figure 2:**
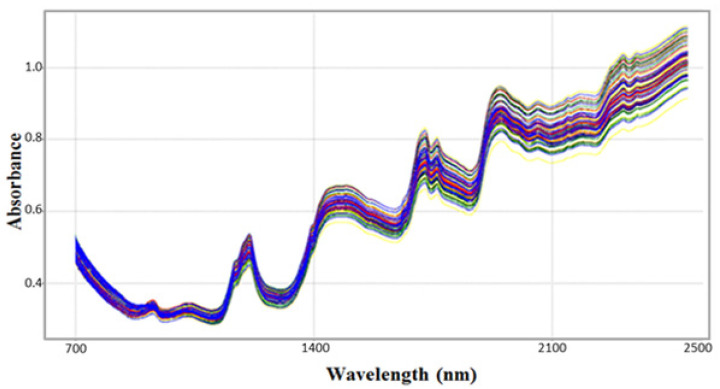
NIR spectra without pretreatment of intact sesame seeds, in the region between 700 and 2500 nm.

The raw spectra (without pretreatment) in the near-infrared (NIR) region of the intact sesame seeds, between 700 and 2500 nm, are represented in Figure 2. There is a band (930 nm), related to the third overtones of the C–H stretching in various groups. There is also a strong band (1200 nm) related to the C–H stretching of the second overtone (-CH2). In approximately 1450 nm, we found an accentuated band, related to the O–H stretching of the first overtone (water) and CO stretching of the third overtone (-CO). At 1500 nm, we found, possibly, a C–H stretching of the first overtone. A strong peak can be observed at 1700 nm, possibly related to the C–O (oil) and C–H stretching of the first overtone (-CH2). At 1765 nm, we found a stretching related to the C–H (oil) and C–H of the first overtone (-CH2). A strong peak can be observed at 1940 nm, possibly related to the O–H bending of the second overtone (water), and at 2300 nm, which is related to the C–H bending of the second overtone (oil). Minor bands are observed at 2060, 2150, and 2180 nm, possibly related to oil and hydrocarbons [29].

It is possible to observe an elevation in the base line and a spreading of the NIR spectra; this is caused by the inhomogeneity of the seeds, that is, by the differences in granulometry, packaging and orientation of the seeds [18,30,31,32]. The different colored lines represent the placed spectra of each sample. Note that due to the overlap it is not possible to perceive the difference between the samples. Therefore, it is necessary to perform pre-processing of the spectral signals in order to remove or soften the spectral noise, the rise in the baseline and the effect of light mirroring due to diffuse reflectance, through some mathematical methods, such as the first and second derivative, SNV (normal signal standardization), MSC (multiplicative signal correction), EMSC (extended multiplicative scatter correction), moving average, Savitzky–Golay, among other techniques and their combinations, for the cultivar discrimination model generated is not biased or models noise [30,31,32].

After observing the good results obtained through the construction of the database for phenotyping and the discrimination of intact seeds of sesame cultivars and strains using infrared spectroscopy in the near region, signal pre-processing techniques and the analysis multivariates PCA and HCA in different databases, we decided to expand the model developed and verify the possibility of authenticating cultivars and strains in the same NIR spectrum bank.

As previously noted, it is almost impossible to distinguish between samples using the raw infrared spectra, and the inhomogeneity of the seeds and differences in particle size and orientation make this distinction even more difficult. Therefore, some mathematical methods were applied before applying the unsupervised pattern recognition techniques, HCA and PCA [33,34].

The dendrogram resulting from the application of the hierarchical cluster analysis—HCA technique that showed the best result was obtained with data treatment centered on the mean, Euclidean metric distance and incremental linkage algorithm, as a grouping rule. It is necessary to first perform smoothing in the light scattering through the application of the standard normal variation transformation—SNV in the spectra.

**Figure 3:**
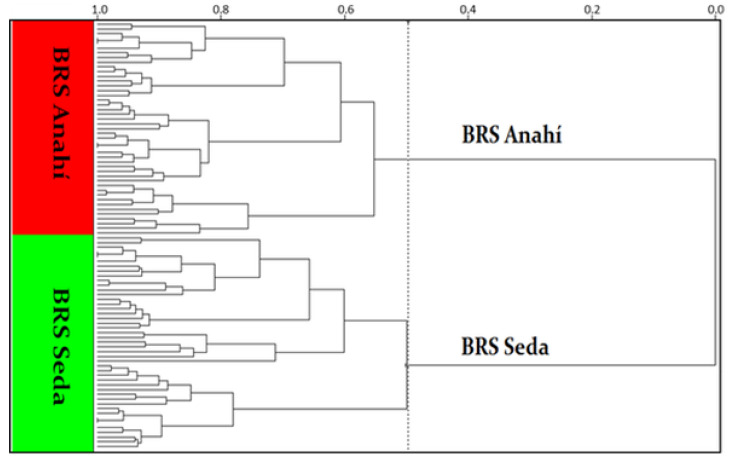
Phenotyping of sesame cultivars BRS Seda (in green) and BRS Anahí (in red), obtained by the HCA technique with data centering on the mean, Euclidean metric distance and incremental connection method associated with SNV + baseline correction, in the spectral region of NIR between 700 and 2500 nm.

Figure 3 shows the dendrogram resulting from the application of hierarchical clustering analysis—HCA to NIR spectral data in intact seeds of sesame cultivars. The technique was successful with the use of spectral treatment, with data centering on the mean, Euclidean metric distance and incremental linkage algorithm, as a grouping rule associated with the application of the standard normal variation transform—SNV associated with baseline correction, considering the full spectrum (700 to 2500 nm). In this figure, it is possible to observe the separation of two large, well-defined groups of the two sesame cultivars, BRS Seda (in green) and BRS Anahi (in red). Researchers performed the geographic discrimination of sesame through chemometric analysis, but this discrimination occurred due to the different chemical compositions in the soil; however, it is possible to discriminate two different sesame cultivars through HCA, grown in the same soil and under the same climatic conditions.

**Figure 4:**
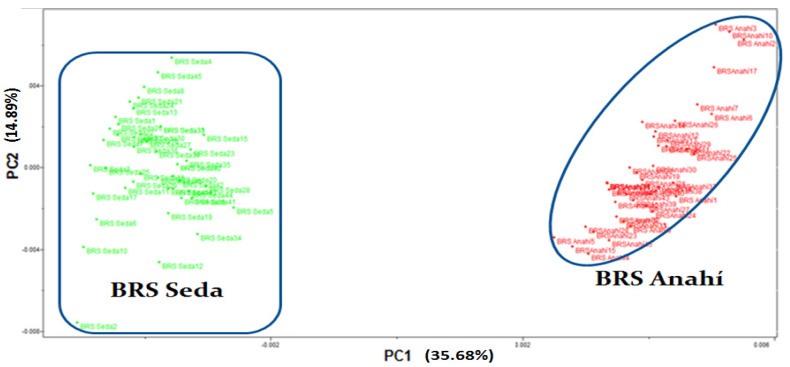
Scores graph for PC1 vs. PC2, which shows the phenotyping of sesame cultivars BRS Seda (in green) and BRS Anahí (in red), using NIR spectra in the region between 700 and 2500 nm, after centering on the mean and applying SNV.

The principal component analysis—PCA technique was also applied, with the use of 2 principal components, which were responsible for describing 50.57% of the total variance, with 35.68% attributed to PC1 and 14.89% to PC2, according to the graph of scores presented in Figure 4, in which the separation of two large groups can be observed, referring to the phenotyping of the sesame cultivars (BRS Seda and BRS Anahí), in accordance with the result obtained by the HCA.

The results obtained corroborate with those in the literature, in which researchers are able to discriminate the seed cultivars, such as soybean, castor beans and rice, through NIR spectroscopy, or what some call NIR phenotyping, because the cultivars have different characteristics from the other existing ones and these must remain for successive generations.

After these observations, we decided to expand the developed model and verify the possibility of authenticating cultivars and strains in the same NIR spectra bank, considering that the strains did not yet have distinct characteristics and therefore did not have the cultivar status.

**Figure 5:**
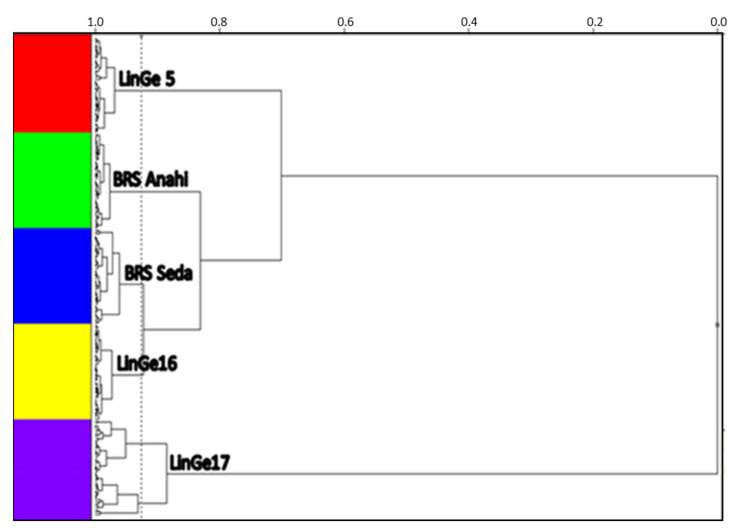
Subsection sesame strains (LinGe5, LinGe16 and LinGe17) and cultivars (BRS Anahí and BRS Seda), using NIR spectra after centering on the mean and applying SNV, using the HCA technique using incremental connection.

The unsupervised pattern recognition model is shown in Figure 5, which demonstrates that when using the spectral data NIR (700 to 2500 nm) in the intact seeds of the sesame cultivars and strains, it is possible to discriminate the samples. It can be observed, through the hierarchical tree, that strain LinGe16 has a high similarity with BRS Seda and that the LinGe5 sesame strain, according to the model, showed 70% similarity with the others (BRS Anahí, BRS Seda and LinGe16).

However, it appears that the samples of the LinGe17 strain did not find similarity with any of the samples of the other strains or cultivars, and, therefore, that the model would be an excellent option, with great potential for identification, including forensics, of that strain within the group of samples presented.

The principal component analysis—PCA technique was also used as an unsupervised pattern recognition method in the spectra of sesame cultivars and strains; the data were centered on the mean and then the SNV technique was applied.

**Figure 6:**
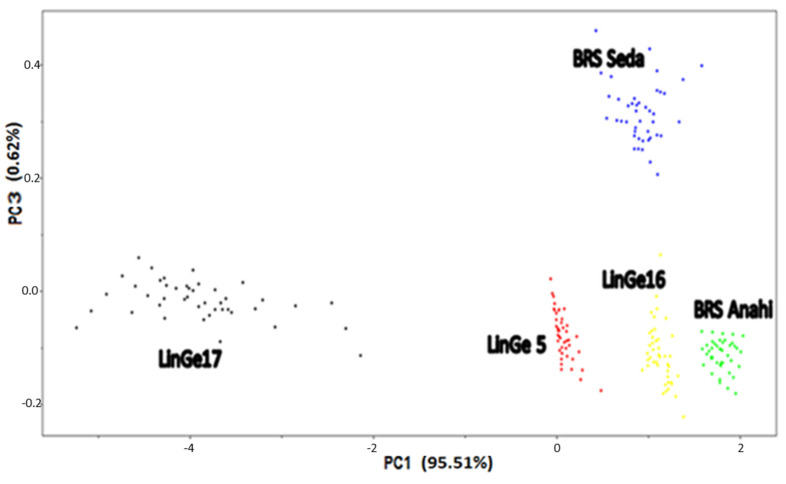
Scores graph for PC1 vs. PC3, which shows the separation of sesame strains LinGe5 (red), LinGe16 (yellow) and LinGe17 (black) l, and sesame cultivars: BRS Anahí (green) and BRS Seda (blue), using NIR spectra in the region between 700 and 2500 nm, after centering on the mean and applying SNV.

Figure 6 presents the graph of scores from PC1vs. PC3, in which it is possible to observe the discrimination of the 5 groups of seeds (the strains Ge5, Ge16 and Ge17 and the cultivars BRS Anahí and BRS Seda), corroborating the result obtained by HCA. PC3 was responsible for describing 0.62% of the total data variance.

Through the application of HCA and PCA, it was possible to observe the discrimination between samples of different sesame cultivars and different sesame strains., As these cultivars and strains were cultivated under the same climatic and soil conditions, we can affirm that it is possible to carry out a “NIR genotyping”, as well as a possible construction of a database for the authentication of the sesame seeds and their respective cultivars and strains, in view of the price variability between them.

After successfully conducting the exploratory analysis of the infrared spectra in the NIR region using unsupervised pattern recognition techniques (principal component analysis—PCA and hierarchical cluster analysis—HCA) for the discrimination of cultivars and strains of the sesame seed, the sesame seed cultivar and strains classification models were constructed using machine learning methods (supervised pattern recognition) using the k-nearest neighbor techniques—KNN and flexible independent modeling by analogy of classes—SIMCA in the studied NIR spectra.

In the calibration set, cross-validation was performed, in which a sample was taken and the model was tested numerous times with the remaining samples. Therefore, it was a parameter/test that proved the calibration adjustment of the model. 

The classification models for sesame seed cultivars and strains were constructed using the supervised pattern recognition techniques KNN and SIMCA in the NIR spectra studied. For testing the models, 30 bottles containing sesame seeds were used in each cultivar (BRS Seda (30 samples) and BRS Anahí (30 samples)) and in each strain (LinGe5 (30 samples), LinGe16 (30 samples) and LinGe17 (30 samples)), totaling 150 spectra for the calibration set and 45 spectra for the external validation set (BRS Seda (15 samples), BRS Anahí (15 samples), LinGe5 (15 samples), LinGe16 (15 samples) and LinGe 17 (15 samples)).

For the construction of the model, pre-processing centering on the mean was used and the technique of multiplicative signal correction (MSC) treatment for the correction of the effect of the scattering of light present in the obtained spectra, was caused mainly due to the lack of optical homogeneity of the samples. The best model obtained is shown in Table 1, in which the results of the prediction for the classification model created for sesame seeds can be observed using KNN, as well as their respective predictions of the samples of the calibration set and of the external validation for the strains (C1(LinGe5), C2(LinGe17) and C3(LinGe13)) and cultivars (C4(BRS Seda) and C5(BRS Anahí)). The cells that are shaded in gray, show the results obtained after the implementation of the model.

The developed KNN model proved to be efficient for the classification of seeds of cultivar and sesame strains, as it correctly classified the 150 samples of the calibration set and the 45 samples of the external validation set, that is, 100% accuracy. These results demonstrate that the construction of a database using machine learning and NIR spectroscopy can make the seed authentication process much faster than traditional methods and much less expensive for companies and research laboratories, as well as for inspection bodies using portable NIRs, as well as being in line with the world policy of sustainable development and green chemistry. 

**Figure 7:**
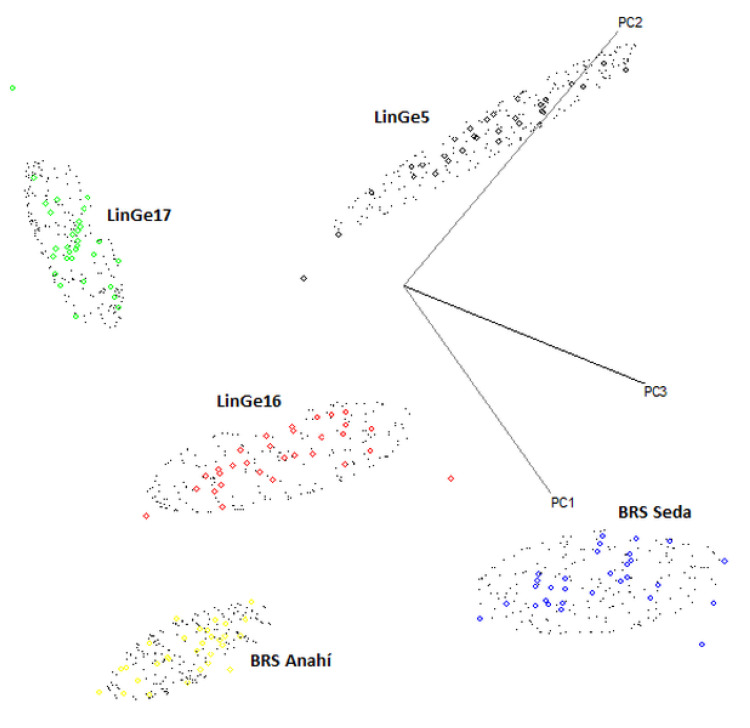
Three-dimensional graphs of PC1 vs. PC2 vs. PC3 of the SIMCA modeling applied to the 150 samples of the calibration set which are classified into 5 classes (strains (LinGe5, LinGe16 and LinGe17) and sesame cultivars (BRS Anahí and BRS Seda)), using NIR spectra, in the region between 700 and 2500 nm, after centering on the average and applying MSC.

Figure 7 shows the three-dimensional plots of PC1 vs. PC2 vs. PC3 that were obtained in the best calibration model of the flexible independent model by class analogy—SIMCA. Strains: LinGe5 (Black), LinGe16 (red) and LinGe17 (green); cultivars: BRS Seda (blue) and BRS Anahí (yellow).

The SIMCA model built using 150 NIR spectrals from the sesame seeds (cultivars BRS Seda (30 samples) and BRS Anahí (30 samples)) and strains (LinGe5 (30 samples), LinGe16 (30 samples) and LinGe17 (30 samples)). 

Was used to predict the 75 sesame seed samples from the external validation set (cultivars BRS Seda (15 samples) and BRS Anahí (15 samples)) and strains (LinGe5 (15 samples), LinGe16 (15 samples) and LinGe17 (15 samples)).

**Figure 8:**
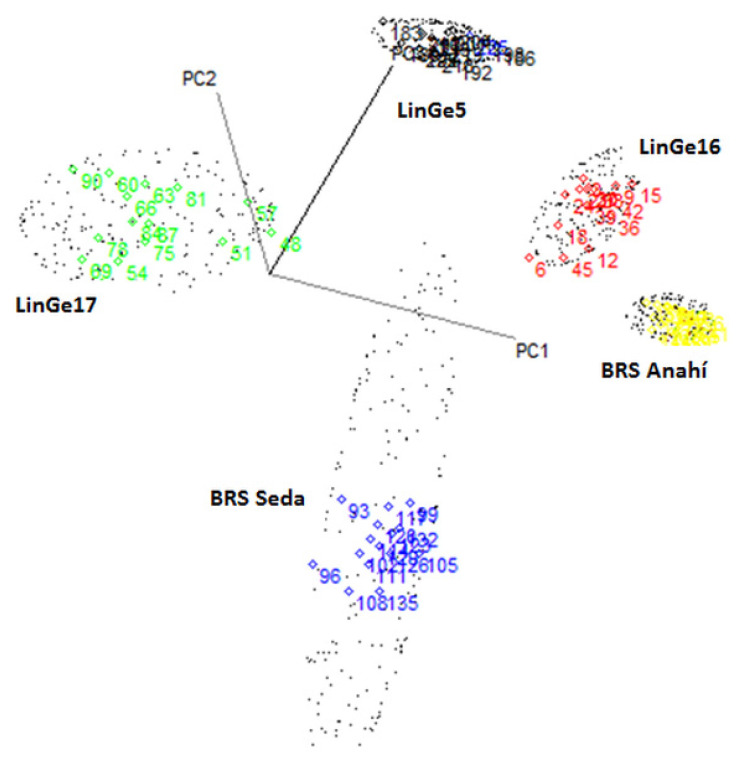
Three-dimensional graphs of PC1 vs. PC2 vs. PC3 of the SIMCA model for the prediction of the classification of the 75 samples of the external validation set, which have the classification of 5 classes (strains (LinGe5, LinGe16 and LinGe17) and sesame cultivars (BRS Anahí and BRS Seda)), using NIR spectra, in the region between 700 and 2500 nm, after centering on the average and applying MSC.

Figure 8 shows the three-dimensional plots of PC1 vs. PC2 vs. PC3, obtained in the prediction of the external validation set for 75 sesame seed samples, through flexible independent modeling by class analogy—SIMCA, after focusing on the mean and applying MSC. Strains: LinGe5 (Black), LinGe16 (red) and LinGe17 (green); cultivars: BRS Seda (blue) and BRS Anahí (yellow).

Table 2 presents the prediction for the classification model created for sesame seeds using the SIMCA technique, as well as their respective predictions of the samples from the calibration and external validation set for strains (C1(LinGe5), C2(LinGe17) and C3(LinGe13)) and cultivars (C4(BRS Seda), C5(BRS Anahí)). The cells that are shaded, in gray, show the accuracy.

The constructed SIMCA model showed efficiency for the classification of the seeds of sesame cultivars and strains, as it correctly classified the 149 of the 150 samples of the calibration set.

Only one sample belonging to the class of LinGe17 was classified incorrectly in the class of cultivar BRS Anahí, that is, 99.3% accuracy. In the external validation model, there was only one error in which a sample belonging to the class of LinGe17 was incorrectly classified as belonging to the class of LinGe16; therefore, 98.7% of accuracy was obtained.

## 4. Conclusions

The technique of infrared spectroscopy in the near region by diffuse reflectance, associated with the unsupervised pattern recognition techniques (principal component analysis—PCA and hierarchical cluster analysis—HCA) and the machine learning techniques (KNN—k-nearest neighbor and SIMCA—flexible independent modeling by class analogy) were able to classify seeds of different sesame cultivars and distinct strains using whole and intact seeds. This was performed in addition to discriminating cultivars and strains of sesame seeds quickly, non-destructively and efficiently, without the use of reagents and the generation of harmful residues, thus preserving the environment. Based on our results, we can determine that our work has great potential to be applied in agriculture and forensic investigations through “NIR genotyping”.

## Figures and Tables

**Table 1:** Predictions of the samples used in the creation of the model and also of the samples from the validation set for the NIR spectra using the KNN.

KNN	Calibration Set (150 Samples)					Validation Set (75 Samples)				
	C1	C2	C3	C4	C5	C1	C2	C3	C4	C5
1	30	0	0	0	0	15	0	0	0	0
2	0	30	0	0	0	0	15	0	0	0
3	0	0	30	0	0	0	0	15	0	0
4	0	0	0	30	0	0	0	0	15	0
5	0	0	0	0	30	0	0	0	0	15

**Table 2:** Prediction of the samples used in the creation of the model and also of the samples from the validation set for the NIR spectra of sesame seeds using the SIMCA technique.

SIMCA	Calibration Set (150 Samples)					Validation Set (75 Samples)				
	C1	C2	C3	C4	C5	C1	C2	C3	C4	C5
1	30	0	0	0	0	15	0	0	0	0
2	0	29	0	1	0	1	14	0	0	0
3	0	0	30	0	0	0	0	15	0	0
4	0	0	0	30	0	0	0	0	15	0
5	0	0	0	0	30	0	0	0	0	15
